# Diagnosis of Morquio Syndrome in Dried Blood Spots Based on a New MRM-MS Assay

**DOI:** 10.1371/journal.pone.0131228

**Published:** 2015-07-06

**Authors:** Claudia Cozma, Sabrina Eichler, Gyula Wittmann, Alba Flores Bonet, Guido Johannes Kramp, Anne-Katrin Giese, Arndt Rolfs

**Affiliations:** 1 Centogene AG, Rostock, Germany; 2 Albrecht-Kossel-Institute, University of Rostock, Rostock, Germany; Roswell Park Cancer Institute, UNITED STATES

## Abstract

**Background:**

Mucopolysaccharidosis IVA (MPS IVA; Morquio A disease) is an autosomal recessive disease caused and characterized by a decreased activity of N-acetylgalactosamine-6-sulfate sulfatase (GALNS), resulting in accumulation of keratan sulfate and chondroitin-6-sulfate in tissues and secondary organ damage. Recently approved enzyme replacement therapy renders the easy and early identification of MPS IVA of out-most importance.

**Methodology:**

We propose a completely new assay for the stable and reproducible detection of GALNS deficiency in dry blood spots (DBS). For the validation blood samples were taken from 59 healthy individuals and 24 randomly selected genetically confirmed MPS IVA patients. The material extracted from DBS was incubated with a 4-methylumbelliferyl-β-D-galactopyranoside-6-sulfate as a specific substrate. Final enzymatic product, 4-methylumbelliferone, obtained after adding exogenous beta-galactosidase, was quantified by LC/MRM-MS (liquid-chromatography/multiple-reaction-monitoring mass-spectrometry). 4-propyl-5-hydroxy-7-methyl-2h-chromen-2-one was used as internal standard, a compound with a similar molecular structure and fragmentation pattern in negative ion mode as 4-methylumbelliferone.

**Findings:**

The enzymatic assay yielded a positive and negative predictive value of 1.0 for genetically confirmed MPS IVA patients (GALNS activity of 0.35 ± 0.21 μmol/L/h) and for controls with normal GALNS activity (23.1 ± 5.3 μmol/L /h). With present enzymatic conditions, the reaction yield in dried blood spots is at least 20 fold higher than any previously reported data with other assays.

**Interpretation:**

The present LC/MRM-MS based assay for MPS IVA diagnosis provides an easy, highly-standardized, accurate and innovative quantification of the enzymatic product in vitro and distinguishes perfectly between MPS IVA affected patients and normal controls. This technique will significantly simplify the early detection of MPS IVA patients.

## Introduction

Mucopolysaccharidoses (MPSs) are a group of inherited lysosomal storage disorders in which defects of different lysosomal enzymes lead to accumulation of glycosaminoglycans in various tissues and organs. Morquio A disease, also known as mucopolysaccharidosis IVA or MPS IVA; [1] is characterized by accumulation of keratan sulfate (KS) and chondroitin-6-sulfate (C6S) at a cellular level in different organs, generating multi-systemic impairments. Keratan sulfate degradation is carried out sequentially in the lysosome where two enzymes are involved in the removal of beta-galactoside moieties: N-acetylgalactosamine-6-sulfatase (GALNS, deficient in MPS IVA) and β-galactosidase (GLB1, impaired in Morquio Type B disease, MPS IVB).

Although some of the clinical symptoms are characteristic for MPS IVA (such as short stature together with progressive skeletal dysplasia), it is nearly impossible to distinguish the two forms of MPS IV based on clinical data alone. Most of the cases present with a common phenotype [2]. MPS IVA (with a prevalence of at least 1 in 200,000 live births, [3]) includes clinical features of skeletal dysmorphism (short stature, dysostosis multiplex, kyphosis, pectus carinatum), corneal clouding, joint contractures, tooth enamel defect and spinal cord compression. The physical signs of MPS IV become distinguishable in the second year of life, while the cognitive functions develop normally.

To date, over 200 GALNS mutations have been described, causing phenotypic heterogeneity which is reflected by varying symptoms and progression of the disorder. A diagnosis of MPS IVA typically follows specific steps: clinical assessment of the symptoms, glycosaminoglycans analysis in urine, GALNS activity determination in patients with high concentrations of urine keratin sulfates, genetic confirmation of the biochemical analysis. At present, clinical treatment of MPS IVA patients varies from management of symptoms to newly developed specific enzyme replacement therapy. State of the art in clinical practice for immediate biochemical screening of MPS IVA patients is based on fluorimetric determination of GALNS activity in fibroblast culture and lymphocytes [4]. These types of assays require a large quantity of biological sample, are time consuming (especially for the skin biopsy culture) and logistically demanding as they require rapid sample processing after sample collection. For these reasons, a viable alternative is urgently needed.

Dried blood spots (DBSs) on standard filter paper as anenzyme source, is already in use for other lysosomal storage diseases [5; 6; 7]. Using whole blood from DBSs in the fluorimetric assays presents several challenges: very low quantities of enzyme, unknown stability of the enzyme in the blood sample, varying preparation and storage conditions in different laboratories, heterogeneity of the blood samples from different persons, interference of blood components with the accuracy of the measurement.

Although there are no clinically available assays for the determination of GALNS activity in DBS, previous publications describe fluorimetric determination of GALNS activity using a modified lymphocyte assay [8] or tandem mass spectrometry based assay using newly developed substrates [9]. Both methods have the advantage of using low amounts of samples; however they both show several shortcomings: imprecise quantification of the product and signal interference due to other blood components in the case of the fluorimetric assay, and in the case of the mass spectrometric assay a low amount of the product is obtained and last but not least, the substrate is not commercially available. Furthermore, a common disadvantage for both assays is the low conversion of the substrates, in the range of 0.1 to 1 μmol/L/h (maximum) for healthy control samples.

Herefore, we present a novel approach in GALNS activity quantification, DBSs based and with a commercially available substrate, improved enzymatic reaction conditions, and a LC/MRM-MS detection and quantification that reduces the matrix effect found in fluorimetric determinations. Moreover, we developed a second enzymatic assay for β-galactosidase to be used as a control for an MPS IVA enzymatic assay using the same MS based detection method.

## Materials and Methods

### Blood samples used in present study

The study and the protocol has been approved by the Ethical Committee of the University of Rostock (Ethics vote #A-2011-109), in accordance with German and European Union legal guidelines. All the participants in the study signed informed consent forms. Dried blood spots (DBS) were prepared from the blood of 59 healthy controls (aged 23 to 61 years); 2 MPS IVA carriers (aged 35 and 35) and 24 randomly selected MPS IVA patients (ages 3 to 19); 4 MPS IVB carriers (aged 27 to 35) and 11 MPS IVB affected patients (aged 3 to 15). All carriers and affected MPS IV patients were genetically confirmed. For the MPS IVA patients, no data are available regarding the KS and C6S levels in urine; however GALNS and beta-galactosidase biochemical assays were performed on leucocyte pellets using an established protocol [4] in parallel with the genetic analysis to confirm the pathogenicity of the samples.

#### Sample preparation

The DBS samples were prepared using ethylenediaminetetraacetic acid (EDTA) blood on filtercards (CentoCard, Centogene AG, Rostock, Germany). Upon preparation, filter cards were dried for 2 hours at room temperature, sealed in zip bags and stored at -20°C until the assays were performed.

#### Chemicals

The substrate used for MPS IVA was 4-methylumbelliferyl-β-D-galactopyranoside-6- sulfate sodium salt (MU-βGal-6S, Glycosynth, Warrington, UK); β-galactosidase exogenous enzyme was necessary for the second reaction step (Protease from Aspergillus oryzae, Sigma-Aldrich, Hamburg, Germany); the substrate for MPS IVB was 4-methylumbelliferyl β-D-galactopyranoside (MU-βGal, Sigma-Aldrich, Hamburg, Germany); as an internal standard, 4-propyl-5-hydroxy-7-methyl-2h-chromen-2-one (Matrix Scientific, South Carolina, USA), was used for all the mass spectrometric analyses of MPS IV assay products. For the control enzyme assay—acid sphingomyelinase—the substrate and internal standards were provided by the Center of Disease Control and Prevention (CDC, Atlanta, USA). The vials contained substrate and internal standard in a molar ratio of about 50:1. Reagents were lyophilized and dissolved in appropriate buffers as previously described [10, 11, 12]. Commercially available salts (Sigma-Aldrich, Hamburg, Germany & VWR, Hannover, Germany) were used for all solutions and buffers.

### Genetic confirmation of MPS IV A/B patients

Genetic analysis was performed on high quality purified DNA. Bidirectional Sanger sequencing of the entire coding region and the highly conserved exon-intron splice junctions was performed with gene and amplicon specific primers. PCR is followed by Shrimp Alkaline phosphatase / exonuclease I treatment; cycle PCR is carried out using BigDye Terminator kit v3.1 (LifeTechnologies) and subsequent ethanol purification. Sequencing was performed using an ABI 3730 x l sequencer. The test has been developed and validated for clinical purposes. The GALNS gene reference sequence is NM_000512.4. The disease causing mutations detected in the GALNS gene of the investigated MPS IVA patients in this study are listed in Table 1. All patients present a MPS IVA phenotype. For all mutations not described in HGMD (or other databases), software analyses have been carried out using Alamut, including SIFT, PolyPhen, MutationTaster and Align GVGD. According to software predictions the novel variants we detected can be considered as likely pathogenic according to prediction software. Large deletions for homozygous variants detected could not be excluded. To confirm the pathogenicity of underlying variants, additional enzyme levels determined in leucocytes using a classical protocol described in literature [4].

**Table 1:** GALNS mutations of patients diagnosed and selected as reference for the MPS IVA enzymatic assay development.

Coding effect	cDNA change	Mutation type	Localization	Allele zygosity	Patient Diagnosis	Ref.
p.Q29X	c.85C>T	Nonsense	exon 1	Homozygous	confirmed	Novel^a^
p.D39Y	c.115G>T	Missense	exon 1	Heterozygous^c^	confirmed	Novel^a^
p.G50R	c.148G>A	Missense	exon 2	Homozygous	confirmed	Novel^a^
p.T100P	c.298A>C	Missense	exon 3	Homozygous	confirmed	Novel^a^
p.A107T	c.319G>A	Missense	exon 3	Homozygous	confirmed	[13; 14]
p.P179S	c.535C>T	Missense	exon 5	Homozygous	confirmed	[13; 14]
p.Y181C	c.542A>G	Missense	exon 5	Homozygous	confirmed	Novel^a^
p.N204T	c.611A>C	Missense	exon 6	Heterozygous^b^	confirmed	Novel^a^
p.F226L	c.676T>C	Missense	exon 7	Heterozygous^b^	confirmed	Novel^a^
-	c.759-3C>G	Splicing	intron 7	Homozygous	confirmed	Novel^a^
p.S287L	c.860C>T	Missense	exon 8	Heterozygous^b^	confirmed	[13; 15;16]
p.T312A	c.934A>G	Missense	exon 9	Homozygous	confirmed	[13; 16,17]
p.G340D	c.1019G>A	Missense	exon 10	Homozygous	confirmed	[13; 14]
p.R386C	c.1156C>T	Missense	exon 11	Heterozygous^b^	confirmed	[13; 18; 19]
p.A392V	c.1175C>T	Missense	exon 11	Homozygous	confirmed	[13; 14]
-	c.1482+1G>A	Splicing	intron 13	Homozygous	confirmed	Novel^a^
p.P498L	c.1493C>T	Missense	exon 14	Homozygous	confirmed	Novel^a^
p.P499L	c.1496C>T	Missense	exon 14	Homozygous	confirmed	Novel^a^

^a^Not described in HGMD, present in CentoMD [20]

^b^Patients carry two heterozygous mutations

^c^Patient carries three heterozygous mutations/variant

### Mucopolysaccharidosis type IVA assay

DBSs with a diameter of 3.2 mm (blood volume ca 3.1 μL) were punched from filter cards. Extraction was performed by adding 20 μL extraction solution (0.5 M sodium chloride, 0.2% deactivated bovine serum albumin, 0,02% sodium azide) and incubating for 70 to 100 minutes at 37°C at 700 rotations per minute (RPM). To the extract, 20 μL reaction buffer 1 (100 mM sodium acetate, 10 mM lead acetate, 2 mM ethylenediaminetetraacetic acid, 0.02% natrium azide, pH 4.5) and 40 μL substrate solution (10 mM MU-βGal-6S, 0.2% deactivated bovine serum albumin, 0.02% natrium azide) were added. Reaction mixture was mixed 10 s with a vortex mixer, sealed and incubated under agitation at 37°C for 44 to 48 h. In a second step, to the reaction mixture 20 μL reaction 2 buffer (100 mM citrate-phosphate buffer, pH 5.2) and 20 μL β-galactosidase solution (10 U/ mL in 0.2% deactivated bovine serum albumin, 0.02% natrium azide) were added. The reaction solution was mixed, sealed and incubated for further 6 h. The enzymatic reaction was stopped by adding 330 μL stop buffer (0.25 M sodium carbonate-bicarbonate buffer with 0.012% Triton x-100, pH 10.7). GALNS activity was determined in duplicates for all samples and controls, different conditions for card preparation, storage and transport were studied to estimate the stability of the GALNS in DBS. Assay validation encompassed experiments such as: intra- and inter-assay accuracy, intra- and inter-assay precision, linearity of the method and of the instrumentation detection, reference values determination with healthy controls and MPS IV A patients, and robustness of the method.

### Mucopolysaccharidosis type IVB assay

Dried blood spots (DBSs) with a diameter of 3.2 mm (estimated blood volume 3.1 μL) were punched from filter cards. Extraction was performed by adding 20 μL extraction solution (0.5 M sodium chloride, 0.2% deactivated bovine serum albumin, 0,02% sodium azide) and incubating for 70 to 100 minutes at 37°C under agitation. To the extract, 40 μL substrate solution (0.8 mM MU-βGal in 100 mM citrate-phosphate buffer, pH 4.4) was added. The reaction mixture was mixed 10 s with a vortex mixer, sealed and incubated under agitation at 37°C for 3 h. The enzymatic reaction was stopped by adding 390 μL stop buffer (0.25 M sodium carbonate-bicarbonate buffer with 0.012% Triton x-100, pH 10.7). The β-galactosidase activity was determined in duplicates for all samples and controls. MPS IVB development was performed using a modified, previously established protocol for β-Galactosidase [8] to be used as control enzyme in routine determinations. Assay validation encompassed experiments such as: intra- and inter-assay accuracy, intra- and inter-assay precision, linearity of the method and of the instrumentation detection, reference values determination with healthy controls and MPS IV B patients, and robustness of the method.

#### MPS IV A and MPS IVB sample clean-up for mass spectrometry analysis

To each assay sample 50 μL internal standard solution (5 μg/mL in methanol) is added, followed by 500 μL organic solvent (ethylacetate: methanol, vol. 19:1). Solution is mixed, centrifuged 3 minutes at 14.5 kRPM. 200 μL from the organic phase are transferred to a 96 well plate with v-shape bottom (Greiner Bio-one, Frickenhausen, Germany), the solvent is evaporated and the analyte- internal standard mixture re-dissolved in 120 μL 10 mM ammonium acetate in methanol: H_2_O, 80:20 v/v.

#### Mass spectrometric analysis of MPS IVA and MPS IVB assays

Mass spectrometric analysis was performed on a triplequad mass spectrometer (TripleQuad 5500, ABSciex, Darmstadt, Germany) coupled with an UPLC system (Water, Manchester, United Kingdom). UPLC was used to concentrate the sample and the internal standard using the following parameters: solvent A—10 mM ammonium acetate in water; solvent B– 10 mM ammonium acetate in methanol; injection volume– 10 μL, flow—0.4 mL/min; gradient—isocratic 80% B; time per analysis—5 min; column—phenomenex fast 4u AAA-MS/ 250 x 2.00 mm, 4 micron. Triplequad mass spectrometer was employed for detection and quantification under the following parameters: type of measurement—multiple reaction monitoring; interface with HPLC—flow splitter (1/3 flow reach the ESI chamber); monitored mass transitions—175/119 (analyte) and 217/160 (internal standard); detection in negative ion mode; resolution Q1 and Q3 –unit; scan time- 4,988 minutes; declustering potential -126V (175/119) and -143 V (162/106); collision energy -38 V (175/119) and -36 V (217/160); curtain gas 40 psi; collision gas 8 psi; ion spray voltage -4000 V; temperature 150°C; ion source gas1 60 psi; ion source gas2 60 psi; entrance potential -12 V; collision cell exit potential: -10 V. Quantification was carried out using 4-propyl-5-hydroxy-7-methyl-2h-chromen-2-one as internal standard (IS) at fix concentration (4 μg/mL) for all samples and a standard curve of 4 –methylumbelliferone (4-MU, analyte).

Statistical data from the MPS IVA and IVB assays validation as well from the control enzyme were analyzed using Excel Software (Microsoft Office, 2010). For each assay and cohort were calculated: minimum, maximum, average, median, standard deviation, cut-off (as average activity minus two times standard deviation). Variations in different measurements and experiments of the same assay were analyzed using ANOVA or Student’s T statistical tests.

### Acid sphingomyelinase assay

3.2 mm spots were incubated at 37°C under agitation for 60 minutes with 80 μL extraction buffer. 10 μL extract were dispensed in 96-well plates. 15 μL of the acid sphingomyelinase (ASM) enzyme assay cocktails were added to the plates containing the extracts, then sealed and incubated for 23 h at 37°C with shaking. The enzyme reaction was stopped with 100 μL of 1:1 ethylacetate and methanol. Sample preparation for MS/MS analysis comprised a liquid-liquid extraction step, a solid phase extraction step and MS/MS detection on an API 4000 triple-quadrupole MS/MS (ABSciex, USA) in positive ion mode using measurement parameters previously described [10].

## Results and Discussion

To evaluate the normal values of GALNS activity in DBS and to calculate the cut-off values, 59 healthy control samples were randomly chosen from our biobank. Filter cards were prepared from freshly collected blood (EDTA blood, 2–6 h old) and from hemolyzed blood (EDTA blood, 2–4 days stored at -20°C). Upon drying, the filter cards were labeled, sealed in zip bags individually, and stored at -20°C to be punched and used for the experiment.

### Development of GALNS enzymatic assay in DBS

GALNS assay development for DBS required experiments to improve each step of the enzymatic assay: (*i*.) extraction (to utilize the small amount of enzyme present to the fullest, reducing enzymatic degradation and instability), (ii.) enzymatic reaction (parameters were tested to boost the enzymatic yield for a better separation of healthy from pathological values), (iii.) sample clean-up, (iv.) detection and quantification of the enzymatic product. Overall, the result of the assay development, GALNS activity in healthy controls was increased from 0.12–0.70 μmol/L/h (previously reported in literature [8]) to 11.0–43.0 μmol/L/h.

**Fig 1:**
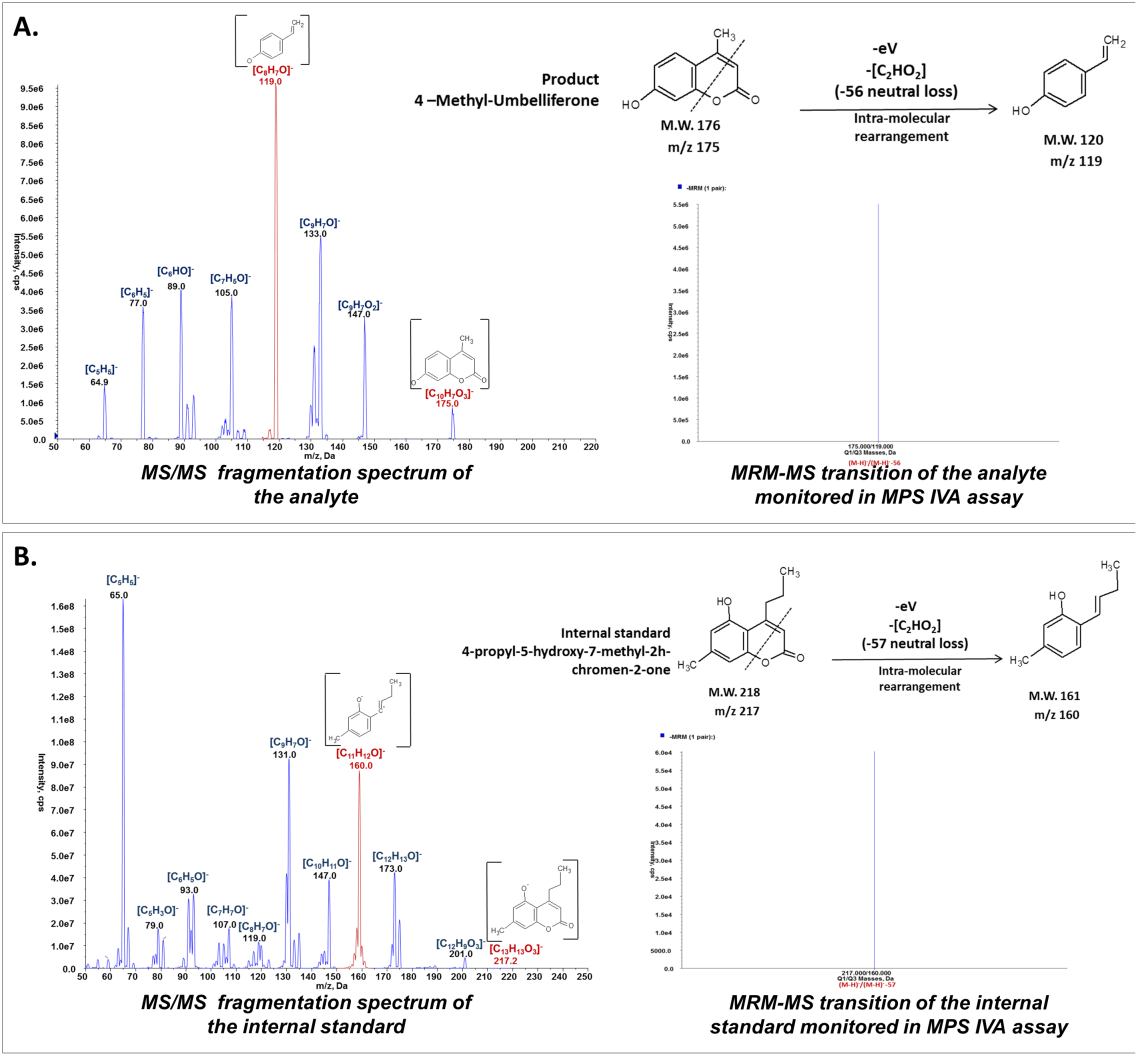
TripleQuad MRM-MS detection of 4-MU. Hydroxyl-chromen-2H-one compounds can be detected in Q1 scan as a (M-H)^-^ ion and, under specific collision energy, the hetero-cycle is broken with a neutral loss of the fragment containing–COO•. We propose that the remaining fragment undergoes a molecular rearrangement to obtain a more stable structure. For enzymatic product detection and quantification, two transitions are monitored: 175/119 (4-MU) and 217/160 (internal standard). **A.** Collision induced dissociation fragmentation spectrum (MS^2^) of the analyte (4-MU), obtained using an ABSciex 5500, and MRM-MS transition spectrum monitored during the MPS IV assays (175/119); B. Collision induced dissociation fragmentation spectrum (MS2) of the internal standard (4-propyl-5-hydroxy-7-methyl-2H-chromen-2-one), obtained using an ABSciex 5500, and MRM-MS transition spectrum monitored during the MPS IV assays (217/160).

1. To develop a viable assay for DBS, first an optimal extraction method of the blood elements from the filter paper was developed, using different buffers and varying pH and concentrations. Out of all the extraction conditions tested, we found that the best results (20% higher than any other extraction solution or buffer tested) were obtained with a concentrated saline solution in the presence of inactivated bovine serum albumin as a stabilizer, under agitation for more than 70 minutes. DBS were incubated at 37°C for a period of 70 to 100 minutes with 20 μL 0.5 M sodium chloride, 0.2% deactivated bovine serum albumin, 0,02% sodium azide (due to the length of the assay incubation times, natrium azide was added to all solutions).
2. GALNS enzymatic activity was found to be linear from 45 to 50 hours for DBS extract. For this reason the incubation time for this step was set to an interval of 46 to 48 h and calculation of the enzymatic activity was adjusted accordingly for each sample. This long period served also to compensate for the low amount of enzyme present in DBS. GALNS activity was found to be enhanced by adding lead (Pb^2+^)—a metal ion that does not easily form adducts with the product in ESI measurement, as well as by the presence of EDTA in the reaction mixture. For the second reaction step, β-galactosidase is diluted with 0.2% bovine serum albumin and the enzymatic reaction is carried out for 6 h.
3. During the sample cleanup step, water soluble elements from the reaction mixture (salts, bovine serum albumin, and hemoglobin) and filter paper rests that can block the LC/MS system are removed by liquid-liquid extraction. The two phases are easily separated (the water phase is reddish due to the blood extract and high salt concentration, the organic is transparent) and the cleanup can be performed either in Eppendorf cups (for low number of samples) or in deep well plate for high throughput.
4. The most important improvement introduced by the present assay, is the development of a stable, reproducible multiple reaction monitoring mass spectrometry method for the detection and quantification of 4-methylumbelliferone (4-MU). Although previous studies on flavonoid compounds refer to 4-MU as an internal standard [21; 22; 23], there are no published studies on the routine quantification of 4-MU by mass spectrometry with an application in clinical chemistry. 4-MU, as other hydroxyl-chromen-2H-one derivatives, is a very stable molecule, fluorescent in UV light and thus used routinely in fluorimetric enzyme assays [24]. However, in the presence of blood elements, especially hemoglobin, fluorescence quenching was reported by different studies. This phenomenon cannot be precisely quantified for each DBS sample separately, rendering a quantification error inevitable. Also, although sensitive, fluorometry uses only a relative quantification through external standard curve. With the development of new instrumentation, multiple reaction monitoring mass spectrometry rivals the sensitivity and the high through-put capacity of the fluorometry; however, by using an internal standard, the selectivity and precision of the quantification is highly superior to the fluorimetric method. Here, we report a new quantification method for 4-MU using an TripleQuad 5500 mass spectrometer (ABSciex, Germany) using a standard curve of nine 4-MU dilutions from 0 μg/mL to 1 μg/mL and an internal standard with a fixed concentration of 0.5 μg/mL. As internal standards for 4-MU quantification, several hydroxyl-chromen-2H-one derivatives were tested and all were found suitable having similar fragmentation pattern with 4-MU under similar collision induced dissociation (CID) conditions. However, 4-propyl-5-hydroxy-7-methyl-2H-chromen-2-one was chosen due to its different parent/daughter transitions (217/160—internal standard and 175/119–4-MU) and similar fragmentation pattern (see Fig 1); also due to its different HPLC retention time compared to 4-MU. The standard curve was linear and reproducible for the concentration range tested (that includes the analytical range for the method).

**Fig 2:**
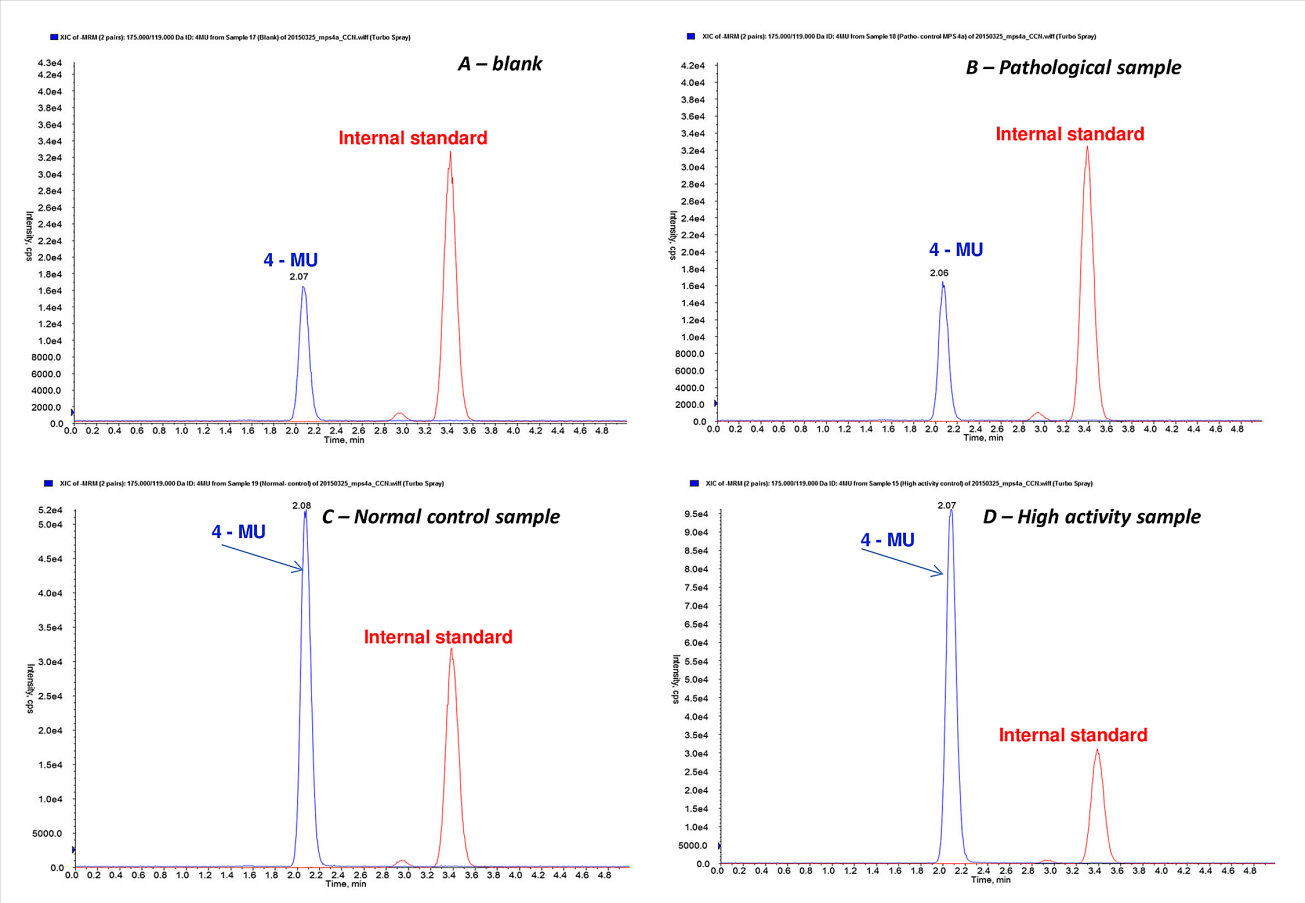
Total Ion Chromatogram profiles of 4-MU at constant concentration of internal standard obtained with TripleQuad MRM-MSfor A—a blank sample or filter paper incubated in the same manner as the blood samples (that contain 4-MU present at the beginning of the enzymatic reaction as a byproduct of the synthesis); B—pathological blood sample (with similar TIC profile as the blank sample, used as a quality control in each assay); C—a normal activity blood sample (healthy control with an average GALNS activity used as a quality control in each assay); D—a high activity blood sample (a sample with atypically high activity of lysosomal enzymes).

For all the MPS 4a tests, the standard curves preparation also incorporated steps that mimic the those used in the preparation of investigated blood samples (dilution in stop buffer, mixing with the internal standard, liquid—liquid extraction) thus measurement differences caused by traces of salts or low extraction yield were eliminated. Examples of TIC (total ion chromatogram) obtained by LC/MRM-MS are shown in Fig 2 for *a blank sample* (A new blank sample is measured in every measurement as background to be subtracted from the values obtained for the samples investigated. This step is necessary due to the free 4-MU present in the substrate before the start of the reaction), *a pathological sample* (similar in profile with the blank sample), *a normal control sample with average GALNS activity*, and *a sample with high lysosomal enzyme activity* (2 x higher than the average).

### Validation of the MPS IV assay

The precision of the assay was characterized by measuring the relative standard deviation in intra- and inter-assay experiments. Intra-assay variation was determined by measuring control samples six times (n = 6) in one single batch, and the results show no significant difference between different aliquots of the sample in the same assay (relative standard deviation between 5.00% and 14.02%). Inter-assay variation was determined by measuring control blood samples and the additional pathological sample on 5 different days (n = 5), on separated batches. The results show no significant difference between aliquots of the sample in the different assays performed on different days (with a relative standard deviation between 8. 4% and 11.5% (see supplementary data).

Standard linearity for the method was established by performing ten times standard curve prepared in similar manner to that of the samples. The results show that 4-MU detection and quantification is linear for concentrations up to 6 μM. The linearity of the enzymatic reaction was checked by using different volumes of extract ranging from 25% to 100% incubated in the same manner (with constant substrate amount).

A limit of detection (LOD, 3*standard deviation) of 0.1 μmol/L blood/h and limit of quantification (LOQ, 10*standard deviation) of 0.3 μmol/L blood/h were determined on blank filter papers measured ten times in the same batch.

The parameter of robustness chosen for this method was the time interval from sample preparation to the mass spectrometry analysis. The ANOVA one way statistical test showed no statistically significant difference between the samples measured up to 48 h after preparation and storage at room temperature, in an evaporation prohibitory environment (plate was covered with aluminum foil and placed into a zip bag).

Normal GALNS values (for healthy controls) in analyzed DBSs are found to be between 11.0 to 43.0 μmol/L blood/h (mean ± standard deviation: 23.1 ± 8.9). Cut-off was set at 5.3 μmol/L blood/h (mean-2*standard deviation).

**Fig 3:**
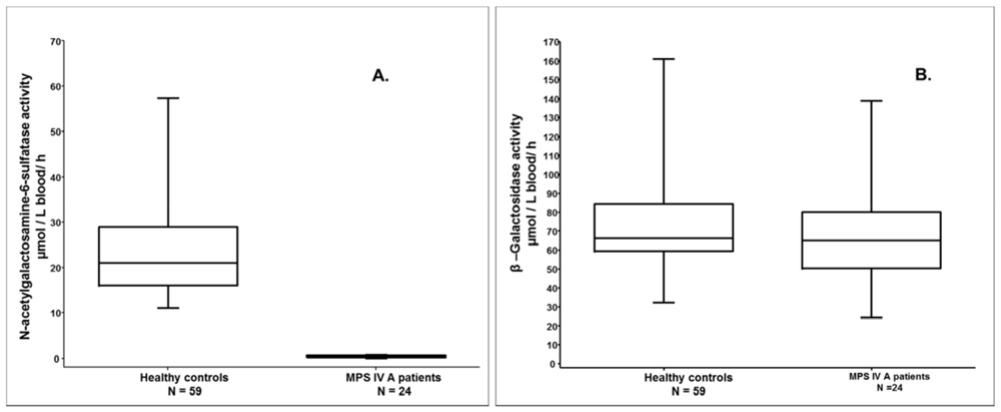
**A. Enzymatic GALNS assay in DBS** shows a statistically significant difference (p<0.0001 in two-tailed Man-Whitney test) between samples of healthy controls and the samples of affected MPS IVA patients. **B. β- galactosidase in DBS**, the proposed control enzyme test, carried out on the control samples and MPS IVA patient samples, show similar activity in both groups.

The pathological range of GALNS activity was determined on 24 MPS IVA patients and determined to be between 0 and 0.64 μmol/L blood /h (mean ± standard deviation: 0.35 ± 0.21)—see Fig 3.

The two MPS IVA carriers showed GALNS activity above the cut off, but lower than the minimum found in DBS from healthy controls (Table 2). Overall positive predictive value, negative predictive value, sensitivity, specificity of the GALNS enzymatic test, calculated for the tested MPS IVA patients and controls, was 1.0 (all MPS IVA cases investigated were correctly assigned as pathological and all controls have demonstrated normal enzyme activity).

**Table 2:** GALNS, GLB1 and ASM enzymatic activities for the investigated MPS IVA patients and carriers, MPS IVB patients and carriers and healthy probands cohort.

		MPS IV A patientsN = 24	MPS IV A carriersN = 2	MPS IV B patientsN = 11	MPS IV B carriersN = 4	Healthy controlsN = 59
**GALNS activityμmol/L/h**	Cut-off	**4.8**				
	Minimum	0.0	6.6	4.7	10.2	11.0
	Maximum	0.6	8.8	20.5	16.1	43.0
	Mean	0.3	7.7	10.9	13.8	22.7
	Median	0.3	7.7	9.0	14.4	20.5
**GLB1 activityμmol/L/h**	Cut-off	**28.5**				
	Minimum	31.6	91.4	0.3	39.5	18.5
	Maximum	132.1	99.4	10.4	58.8	160.9
	Mean	66.6	95.4	3.3	49.3	70.9
	Median	65.0	95.4	3.7	49.4	66.1
**ASM activityμmol/L/h(control enzyme)**	Cut-off	**2.2**				
	Minimum	3.6	11.9	3.7	6.8	4.0
	Maximum	35.2	7.4	24.8	22.3	44.2
	Mean	8.0	9.6	13.0	11.3	8.9
	Median	5.4	9.6	3.0	8.0	8.2

Normal β-galactosidase values in analyzed DBSs from the healthy controls were found to be in the interval 71.8 to 160.9 μmol/L blood/h (alongside an outlier of 18.5 μmol/L blood/h—below the cutoff obtained on healthy samples, but greatly increased compared with the pathological range), with a cut-off of 28.5 μmol/L blood/h. Pathological β-galactosidase activity in DBS samples from the MPS IVB patients was 0.4 to 10.4 μmol/L blood/h, while in the DBS from MPS IVA patients was determined to be 24.2 to 132.1 μmol/L blood/h (normal β-galactosidase activity)—see Fig 3. The 4 carriers investigated presented activities lower than the minimum healthy control, but higher than the cut-off (see Table 2). β—galactosidase enzymatic test has a positive predictive value of 0.93, negative predictive value and sensitivity 1.0 and specificity 0.99. The second enzyme provides a control of the DBS stability and excludes the possibility of a MPS IVB diagnosis. GALNS assay is not sufficient to diagnose with a probability of 100% MPS IVA patients. In the case of low GALNS activity we recommend the investigation of a second sulphatase to exclude the diagnosis of multiple sulphatase deficiency and, when possible, the genetic confirmation of the diagnosis (since any enzymatic assay test is only a screening method).

To confirm the validity of the samples using an established assay for DBS, a third enzyme activity was investigated—acid sphingomyelinase. The measurement showed that all blood samples used in the present study were enzymatically active had an ASM activity greater that the cut-off (2.2 μmol/L/h, calculated as mean-2*standard deviation of the ASM activity for the healthy controls).

#### Quality parameters in daily routine and limitations of the assay

In daily routine several quality parameters were introduced to assert and manage the quality of the samples received by our laboratory: (i.) cumulative time at room temperature (including transport) from filter card preparation to the analysis at a maximum of 10 days; (ii.) upon arrival samples were registered and stored at 4°C until analysis in zip bags (iii.) short turnaround time of 7 days maximum (iv.) the quality of the sample is asserted by using beta-galactosidase as a control enzyme and when its activity is low, a complete multiplex assay is performed as previously described [10]- this eliminates the possibility of pre-analytical deactivation of the enzymes in DBS due to sample preparation and storage; (v.) samples are always analyzed in duplicates from two different spots to verify their homogeneity; (vi.) a blank (a clean filter cards punch) is prepared with each batch in the same manner and with the same buffers as the sample; the results from the blank are subtracted from the values obtained for samples (the substrate contains free 4-MU in different quantities that must be subtracted to obtain an accurate result of the enzymatic reaction); (vii.) quality control samples with known GALNS activity (upper and lower limit statistically determined from 10 different aliquots) are incubated with each batch (a sample from a MPS IVA patient, a normal control with average GALNS activity, in laboratory obtained standard blood samples with zero, low-, medium- and high enzymatic activity)- to assert the quality of the determination; (viii.) with each batch a 4-MU standard curve prepared in the same manner as the samples is measured and used for quantification to eliminate any errors in the LC/MRM-MS analysis that can occur from one measurement to another (e.g. column usage, solvent variation).

**Fig 4:**
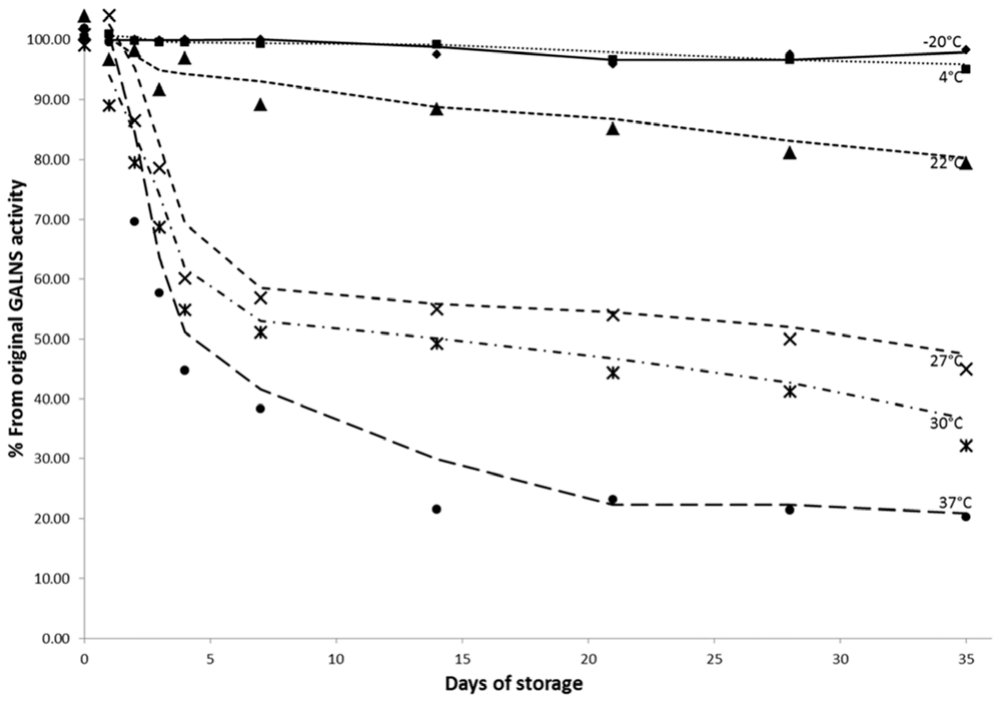
Influence of storage conditions on GALNS activity in DBS. Stability tests show rthat filtercards with DBS stored in zip bags maintain their enzymatic activity when stored at– 20°C and 4°C up to 35 days, while samples stored at a temperature of 27°C and above lose over 40% of their activity in the first 5 days. Ideal sample transport should be at a maximum of 22°C and storage at 4°C or below.

Compared with other lysosomal enzymes that are stable for longer time periods (months) in DBS stored at room temperature, our experiments showed that *GALNS activity is dependent on the transport and storage conditions* of the filtercards. Stability tests performed on the same blood sample at -20°C, + 4°C, room temperature, and 37°C, on sealed and air exposed filtercards, showed a decrease of enzymatic activity in the unsealed filtercards compared with the ones stored in plastic bags for the same temperature. The same study proved stability of GALNS activity for the samples stored at -20°C and at 4°C for at least 6 weeks in sealed plastic bags (Fig 4).

The design of the present MPS IVA assays was preferred as a single assay. Multiplexing (measuring the enzymatic of more than one assay in a single measurement) was excluded primarily due to the length of incubation time in the first step (46 to 48 h), which is th longest compared to incubation steps from other lysosomal enzymes (in our experience with other enzymes from DBS the maximum incubation time was 24h). Secondly, the substrate used is commercially available as 4-MU derivative and for possible multiplexing a chemical modification in the 4-MU moiety is necessary.

This study was performed on a limited number of patient samples (n = 24) with ages from 3 to 19 years and the severity of the GALNS deficit on the investigated samples is not age related. There is no information about the GALNS activity in DBS from newborns at the present moment, another study with a large number of DBSs from newborns is in planned.

## Conclusion

We developed a specific assay for MPS IVA that distinguishes between healthy controls and affected individuals with a negative predictive value, positive predictive values, sensitivity and specificity of 1,0. As a control for the MPS IVA assay, a second assay was developed for beta-galactosidase.

## Supporting Information

S1 File Clinical and genetic data for the MPS IVA patients used in present study. (PDF)

S2 File Influence of free 4-MU in the substrate on the outcome of the enzymatic assay. (PDF)

S3 File Influence on the hemolysis of the EDTA blood in the GALNS assay in DBS. (PDF)

S4 File Summary of the GALNS assay validation. (PDF)
