# Supplementary material for: Diagnosis of Morquio Syndrome in Dried Blood Spots Based on a New MRM-MS Assay
Source: PLoS One. 2015 Jul 6;10(7):e0131228. doi: 10.1371/journal.pone.0131228 (PMC4492791; doi:10.1371/journal.pone.0131228)
Supplement: S1 File — (PDF) [file pone.0131228.s001.pdf]

S1 Table. Clinical and genetic data for the MPS IVA patients used in present study: age, gender, genetic diagnosis, GALNS and beta-galactosidase (beta-Gal.) activity in leucocytes obtained using van Diggelen protocol [4.]. All the MPS IVA patients presented clinical symptoms consistent with MPS IV phenotype, while the carriers presented no clinical symptoms that indicated the presence of MPS IV.Sequence Reference for GALNS NM_000512.4.

MPS IVA Patient	Age	Gender	Coding effect	cDNA change	Mutation type	Localization	PolyPhen-2	Reference	Genotype	In vitro enzymatic test in leucocytes nmol MU/mg protein		
										GALNS[a]	Result GALNS enzymatic test	beta-Gal.[b]
patient 1	19	female	p.(?)	c.759-3C>G	Splicing	intron 7		novel, CentoMD®	Homozygous	< 1	pathologic	861
patient 2	4	male	p.T312A	c.934A>G	Missense	exon 9	probably damaging	Wang (2010) J HumGenet 55:534	Homozygous	< 1	pathologic	490
patient 3	10	male	p.Y181C	c.542A>G	Missense	exon 5	probably damaging	novel, CentoMD®	Homozygous	3	pathologic	797
patient 4	10	male	p.G50R	c.148G>A	Missense	exon 2	probably damaging	novel, CentoMD®	Homozygous	< 1	pathologic	520
patient 5	6	male	p.(?)	c.1482+1G>A	Splicing	intron 13		novel, CentoMD®	Homozygous	< 1	pathologic	313
patient 6	4	male	p.P498L	c.1493C>T	Missense	exon 14	probably damaging	novel, CentoMD®	Homozygous	< 1	pathologic	890
patient 7	6	female	p.G340D	c.1019G>A	Missense	exon 10	probably damaging	Tomatsu (2004) J Med Genet 41:e98	Homozygous	< 1	pathologic	563
patient 8	8	female	p.G340D	c.1019G>A	Missense	exon 10	probably damaging	Tomatsu (2004) J Med Genet 41:e98	Homozygous	< 1	pathologic	405
patient 9	6	male	p.P498L	c.1493C>T	Missense	exon 14	probably damaging	novel, CentoMD®	Homozygous	< 1	pathologic	476
patient 10	8	male	p.A107T	c.319G>A	Missense	exon 3	probably damaging	Tomatsu (2004) J Med Genet 41:e98	Homozygous	< 1	pathologic	620
patient 11	5	female	p.T100P	c.298A>C	Missense	exon 3	probably damaging	novel, CentoMD®	Homozygous	2	pathologic	830
patient 12	9	female	p.G340D	c.1019G>A	Missense	exon 10	probably damaging	Tomatsu (2004) J Med Genet 41:e98	Homozygous	< 1	pathologic	1063
patient 13	na	male	p.P179S	c.535C>T	Missense	exon 5	probably damaging	Terzioglu (2002) Hum Mutat 20:477	Homozygous	5	pathologic	613
patient 14	na	male	p.P179S	c.535C>T	Missense	exon 5	probably damaging	Terzioglu (2002) Hum Mutat 20:477	Homozygous	< 1	pathologic	963
patient 15	7	male	p.P498L	c.1493C>T	Missense	exon 14	probably damaging	novel, CentoMD®	Homozygous	< 1	pathologic	573
patient 16	na	male	p.P498L	c.1493C>T	Missense	exon 14	probably damaging	novel, CentoMD®	Homozygous	< 1	pathologic	596
patient 17	3	male	p.Q29X	c.85C>T	Nonsense	exon 1		novel, CentoMD®	Homozygous	< 1	pathologic	777
patient 18	na	female	p.T312A	c.934A>G	Missense	exon 9	probably damaging	Wang (2010) J HumGenet 55:534	Homozygous	< 1	pathologic	810
patient 19	8	female	p.A392V	c.1175C>T	Missense	exon 11	probably damaging	Tomatsu (2004) J Med Genet 41:e98	Homozygous	< 1	pathologic	842
patient 20	12	male	p.F156L	c.466T>C	Missense	exon 4	probably damaging	Morrone (2014) Mol Genet Metab 112:160	Homozygous	< 1	pathologic	1034
patient 21	4	male	p.S287L	c.860C>T	Missense	exon 8	probably damaging	Bunge (1997) Hum Mutat 10:223	Compound	< 1	pathologic	1003
			p.R386C	c.1156C>T	Missense	exon 11	probably damaging	Ogawa (1995) Hum MolGenet 4:341	heterozygous			
patient 22	6	male	p.S287L	c.860C>T	Missense	exon 8	probably damaging	Bunge (1997) Hum Mutat 10:225	Compound	< 1	pathologic	920

			p.R386C	c.1156C>T	Missense	exon 11	probably damaging	Ogawa (1995) Hum MolGenet 4:341	heterozygous			
patient 23	8	male	p.D39Y	c.115G>T	Missense	exon 1	probably damaging	novel, CentoMD®	Other	< 1	pathologic	720
			p.N204T	c.611A>C	Missense	exon 6	probably damaging	novel, CentoMD®	complex			
			p.F226L	c.676T>C	Missense	exon 7	benign	novel, CentoMD®				
patient 24	11	female	p.D39Y	c.115G>T	Missense	exon 1	probably damaging	novel, CentoMD®	Other	< 1	pathologic	630
			p.N204T	c.611A>C	Missense	exon 6	probably damaging	novel, CentoMD®	complex			
			p.F226L	c.676T>C	Missense	exon 7	benign	novel, CentoMD®				
carrier 1	36	female	p.N204T	c.611A>C	Missense	exon 6	probably damaging	novel, CentoMD®	Other	na	na	na
			p.F226L	c.676T>C	Missense	exon 7	benign	novel, CentoMD®	complex			
carrier 2	36	male	p.D39Y	c.115G>T	Missense	exon 1	probably damaging	novel, CentoMD®	Heterozygous	na	na	na

na = data not available

[a]Normal values for GALNS activity in leucocytes are between 107-198 nmol MU/mg protein

[b]Normal values for beta -Galactosidase activity in leucocytes are between 119-65
