# Supplementary material for: Diagnosis of Morquio Syndrome in Dried Blood Spots Based on a New MRM-MS Assay
Source: PLoS One. 2015 Jul 6;10(7):e0131228. doi: 10.1371/journal.pone.0131228 (PMC4492791; doi:10.1371/journal.pone.0131228)
Supplement: S2 File — (PDF) [file pone.0131228.s002.pdf]

S2. Influence of free 4-MU in the substrate on the outcome of the enzymatic assay.

Commercially available substrates for MPS IVA were determined to contain up to 0.2 % free 4-MU. The percentage of free 4-MU is different not only from vendor to vendor, but also from lot# to lot# from the same vendor. The following experiments were carried out to determine the influence of the free 4-MU on the sensitivity of the assays:

(i.) Substrate solutions spiked with 4MU (0 µg/mL, 0.05 µg/mL, 0.1 µg/mL, 0.5 µg/mL, 1 µg/mL , 2 µg/mL and 5 µg/mL) were analyzed with the same LC-MRM-MS method used for the MPS IV assays. From all the results were subtracted blank (0% 4-MU added) and the recovery rates were found to be between 98,0 and 100.5 %.

No.Crt.	Added 4MU (µg/mL)	Experimental concentration	Recovery rate (Exp. Conc.-Concsol 1/ added 4MU*100)
1	0	1.03	na
2	0.05	1.08	100.0
3	0.1	1.12	90.0
4	0.5	1.52	98.0
5	1	2.03	100.0
6	2	3.04	100.5
7	5	6.02	99.8

(ii.) Substrate solutions, one spiked with 4-MU 25 µg/mL (the equivalent of 1 µg per sample) and one prepared without extra 4-MU in the assay were used to determine the GALNS activity in a blank and in a control sample.

	Assay prepared without extra 4MU Average (n=3)			Substrate solution with added 4MU Average (n=3)		
	Blank	Control sample	Sample - Blank	Blank	Control sample	Sample-Blank
GALNS activity (µmol/L/h)	1.2	18.0	*16.8*	21.3	37.9	*16.7*

As a result of the above experiments, a blank sample (1 punch of clean filter paper, incubated) was added to all the batches analyzed in oue laboratory. The blank was prepared exactly in the same manner, using the same substrate solution as the analyzed samples and the result obtained in the blank sample is subtracted from the results of the analyzed samples. Moreover, samples from a MPS IVA patient and from a healthy control with known GALNS are measured in each experiment as quality controls.
