# Supplementary material for: Diagnosis of Morquio Syndrome in Dried Blood Spots Based on a New MRM-MS Assay
Source: PLoS One. 2015 Jul 6;10(7):e0131228. doi: 10.1371/journal.pone.0131228 (PMC4492791; doi:10.1371/journal.pone.0131228)
Supplement: S3 File — (PDF) [file pone.0131228.s003.pdf]

S3. Influence of the EDTA blood hemolysis on the GALNS assay in DBS

Filter cards were prepared using fresh blood (0-4 h old) and hemolyzed blood (blood was stored at -20°C for 2 days, thawed at room temperature and used for filter cards). The filter cards, upon drying, were stored at -20 °C. MPS IVA was performed and the values were statistically analyzed using Student's T-test. The results are summarized in the S3.Table and visualized in S3. Fig in parallel with the pathological values. The statistical analysis shows no significant difference between the measurements performed with fresh blood and hemolyzed blood.

S3.Table .GALNS activity in fresh blood vs. hemolyzed blood

	Fresh blood samples (N=56)	Hemolyzed blood samples (N=57)
**Min**	11.0	11.4
**Max**	43.0	41.1
**Mean**	23.1	21.1
**STD**	8.9	6.5
**CUT OFF (Mean-2*STD)**	5.3	8.1

Unpaired T test

**P value**	0.1781
**P value summary**	no significance
**Are means signif. different? (P < 0.05)**	No
**One- or two-tailed P value?**	Two-tailed
**Mean ± SEM of column A**	23.08 ± 1.190; N=56
**Mean ± SEM of column B**	21.09 ± 0.8622; N=57
**Difference between means**	1.986 ± 1.465
**95% confidence interval**	-0.9201 to 4.891
**R squared**	0.01627

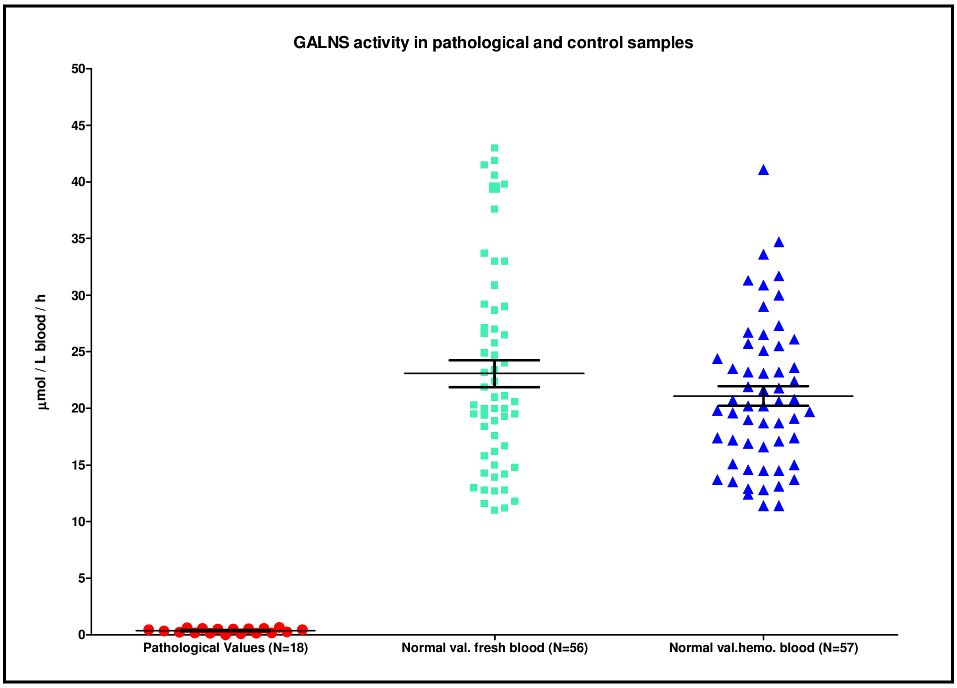

S3 Fig. GALNS activity in fresh blood vs. hemolyzed blood
