# Supplementary material for: Diagnosis of Morquio Syndrome in Dried Blood Spots Based on a New MRM-MS Assay
Source: PLoS One. 2015 Jul 6;10(7):e0131228. doi: 10.1371/journal.pone.0131228 (PMC4492791; doi:10.1371/journal.pone.0131228)

S4. Summary of the GALNS assay validation

(i.) Intra-assay variation (within-run precision)

Intra-assay variation was determined by measuring standard blood samples (low activity standard; medium activity standard; high activity standard) 6 times in a single batch. The results, summarized in S4.1.Table, show that relative standard deviation or RSD % (variability coefficient) was <15% for samples with enzymatic activity. The experiment concluded that there is no significant intra-assay variation.

Intra assay variation results for standard blood samples

standard blood sample N=6	mean activity µmol/L/h	std	rsd %
Low activity standard	3.36	0.17	5.10
Medium activity standard	11.68	1.63	14.02
High activity standard	17.47	1.55	8.85

std - standard deviation; rsd – relative standard deviation

(ii.) Inter assay variation (between-run precision)

Inter-assay variation was determined by measuring the standard blood samples (low activity standard; medium activity standard; high activity standard f), 1 pathological sample and 1 normal control in 5 different days, in separate batches. The results, summarized in S4.2.Table, show no significant inter-assay variation between aliquots of the same sample with RSD% < 15%.

Inter assay variation results for standard blood samples

blood sample N=5	mean activity µmol/L/h	std	rsd (%)
Low activity standard	3.94	0.38	9.6
Medium activity standard	11.67	1.06	9.1
High activity standard	18.14	2.08	11.5
MPS IV a patient	0.64	0.07	10.9
Normal Control*	15.24	1.28	8.4

***Normal control is here defined as a blood sample from a healthy individual with normal GALNS activity**
std - standard deviation; rsd – relative standard deviation

(iii.) Specificity of the method

MRM-MS transitions monitored in MPS IVa assay

No	Analyte	Unique transition to be monitored
1	4- MU	175.0/119.0
2	Internal standard	217.0/160.0

Matrix effect on the MS analysis was investigated in 5 routine determinations. The ratio of the IS intensity in blank (filter paper, no blood) to the IS intensity in pathological control sample (containing blood matrix). The ratio of the IS in blank vs. blood samples was found to be 1.0 +/-0.1 showing no matrix interference.

Matrix interference in the MPS IVA determinations

	$M_{IS}DBS$: $M_{IS}B$
Mean (N=5)	1.0
std	0.13
rsd%	13.6
std - standard deviation; rsd – relative standard deviation	

(iv.) Accuracy of the determination

Within- and between-run accuracy of the determination

Within run accuracy (N=6)			
Nominal Concentration ng/mL	Mean Accuracy	std	rsd%
2.4	105.7	15.9	15.0
1050.0	99.3	3.6	3.6
2250.0	100.2	2.0	2.0
4400.0	99.8	3.3	3.3
Between run accuracy (N=6, E=2)			
Nominal Concentration	Mean Accuracy	std	rsd%
2.4	105.8	0.2	0.2
1050.0	99.1	0.3	0.3
2250.0	100.0	0.3	0.3
4400.0	99.2	0.9	0.9
N=no of aliquots pro sample, E=no of experiments performed			

(v.) Standard linearity of the 4-MU

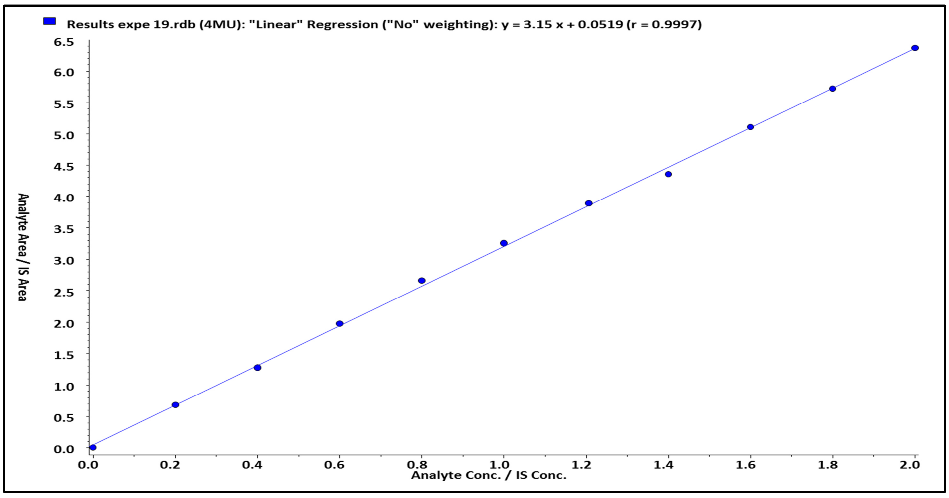

Standard curve performed with solutions obtained directly by diluting a concentrated solution of the analyte - 4MU.

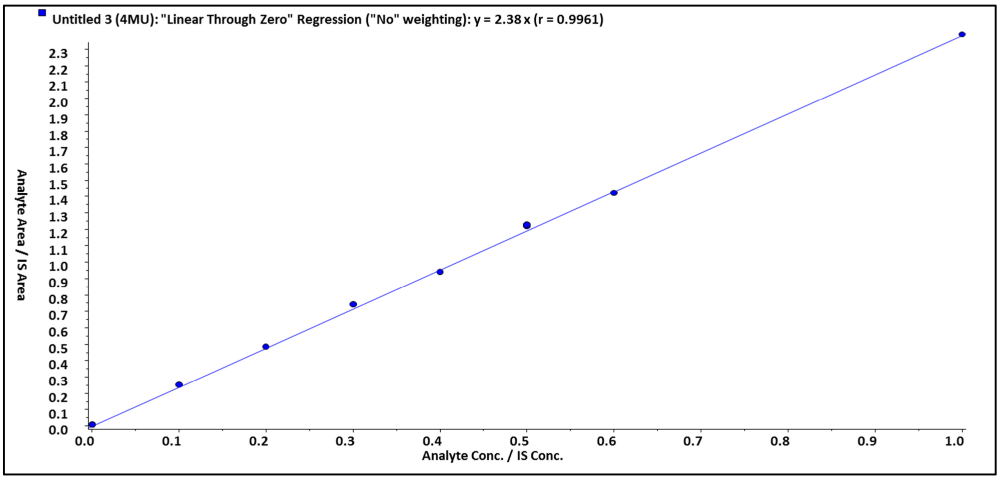

Standard curve performed on each measured plate with solutions prepared in the same manner as the blood samples (dilution in reaction buffers, liquid-liquid extraction, evaporation, dissolving in HPLC solvents).

(vi.) Normal control reference values

GALNS activity in DBS from normal controls samples

	GALNS activity µmol/L/h
Number of samples (n=57)	
minimum	11.0
maximum	43.0
mean	23.1
standard deviation	8.9
CUT OFF (mean-2*std)	5.3

(vii.) GALNS activity in pathological samples

GALNS activity in DBS from MPS IVA patients

	GALNS activity µmol/L/h
Number of samples (n=24)	
minimum	0.00
maximum	0.64
mean	0.35
standard deviation	0.21

Case study: – Case of a family with MPS IVA patients: both parents are carriers but not affected and the two children both with severe symptoms of Morquio A syndrome. DBSs were obtained from fresh EDTA blood, and on these samples GALNS activity was measured using the protocol described in part 2. The results show a very reduced GALNS activity in DBSs from the children, while the DBSs from the parents present normal GALNS activity.

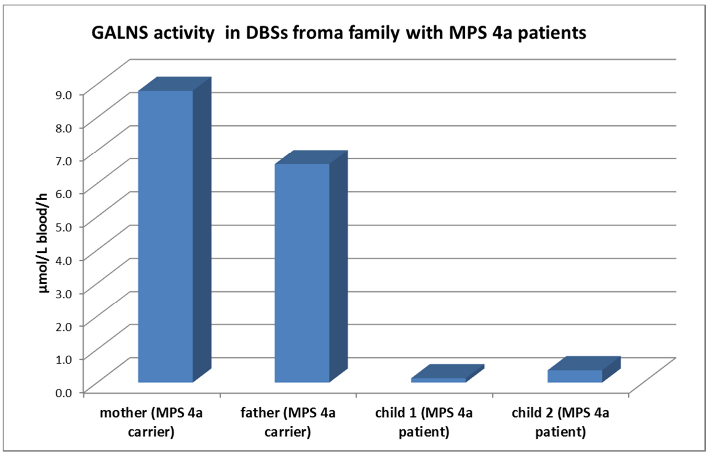

GALNS activity in DBS from a family with MPS IVA patients

(viii.) NPV and PPV

		Patients with MPS4a (as confirmed on molecular genetics)		
		Condition positive	Condition negative	
MPS 4a enzymatic assay	Test outcome positive	**True positive** (TP) = 24	**False positive** (FP) = 0	Positive predictive value = TP / (TP + FP) =1
	Test outcome negative	**False negative** (FN) = 0	**True negative** (TN) = 57	Negative predictive value = TN / (FN + TN) =1
		Sensitivity = TP / (TP + FN) =1	Specificity = TN / (FP + TN) =1	

(ix.) Limit of detection

LOD and LOQ of GALNS enzymatic product in DBS

**Blank control (N=10)**	**GALNS activity (µmol/L/h)**
**Standard deviation**	**0.03**
**LOD (3*standard deviation)**	**0.1**
**LOQ (10*standard deviation)**	**0.3**

(x.) Robustness of the method

Results of the robustness experiment samples – stability of the samples after clean-up (plates covered, at room temperature for 0 h, 24 h and 48 h)

		Base pool	Low activity standard	Medium activity standard	High activity standard
0 h	Mean	0.65	2.54	11.22	19.67
	Std. Deviation	0.50	0.18	3.00	3.42
	Std. Error	0.20	0.07	1.22	1.40
24 h	Mean	0.30	2.42	10.04	16.80
	Std. Deviation	0.16	0.27	1.71	2.62
	Std. Error	0.06	0.11	0.70	1.07
48 h	Mean	0.28	2.66	11.71	22.43
	Std. Deviation	0.12	0.51	0.82	4.20
	Std. Error	0.05	0.21	0.34	1.71
**ANOVA –One way analysis of variance**					
P value		0.098	0.530	0.376	0.413
P value summary		ns	ns	ns	ns
Are means signif. different? (P < 0.05)		No	No	No	No
Number of groups		3	3	3	3
F		2.718	0.664	1.046	0.939
R squared		0.266	0.086	0.122	0.111

ns = no significance; P = permutation values; F = distribution with degrees of freedom; R^2 = multiple correlation coefficient squared

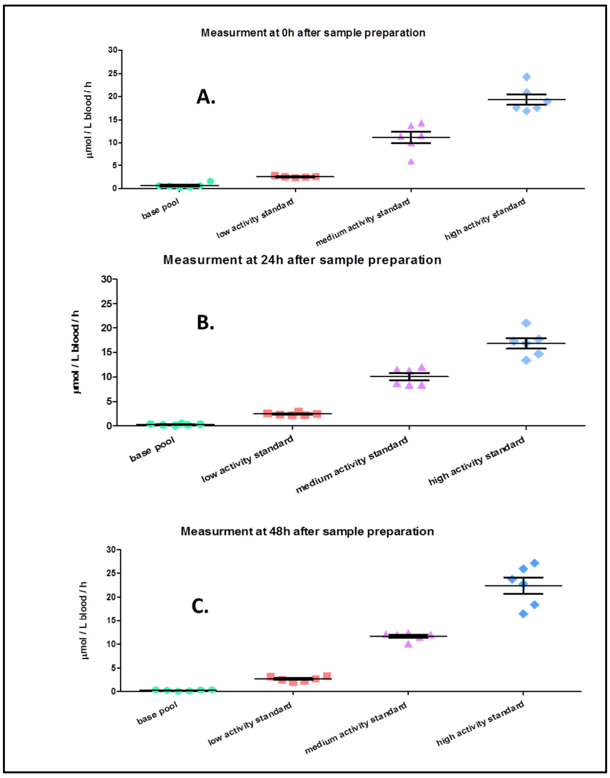

Robustness of the assay – basepool, low, medium and high activity blood samples measured at 0 h, 24 h and 48 h after sample preparation

(xi.) Linearity of the GALNS enzymatic activity

Linearity of the enzymatic reaction using standard blood samples: Standard blood samples were obtained in Centogene Laboratories using a blood sample with high activity of the lysosomal enzymes according with *de Jessus et al. (2009)*: basepool (0% activity from the original enzymatic activity); low activity standard (5% activity from the original enzymatic activity); medium activity standard (50% activity from the original enzymatic activity); and high activity standard (100% activity from the original enzymatic activity).

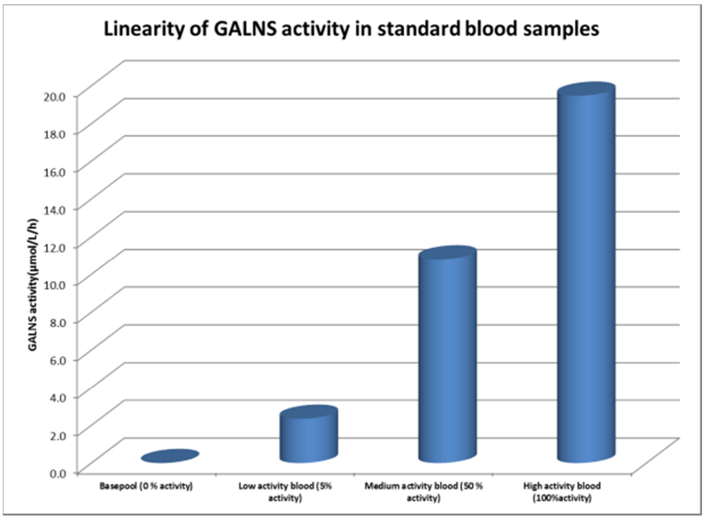

Linearity of the enzymatic reaction using different volumes of DBS extract (different amount of sample): 4 different punches from the same sample were subjected to extraction .Volumes corresponding to 0 %, 25 %, 50 %, 75 %, and 100 % of normal volume of sample were further used for the next steps of the assay, brought to normal volume with 0,2% BSA solution . The volume of sample and the results of the enzymatic assay were plotted and found to be linear

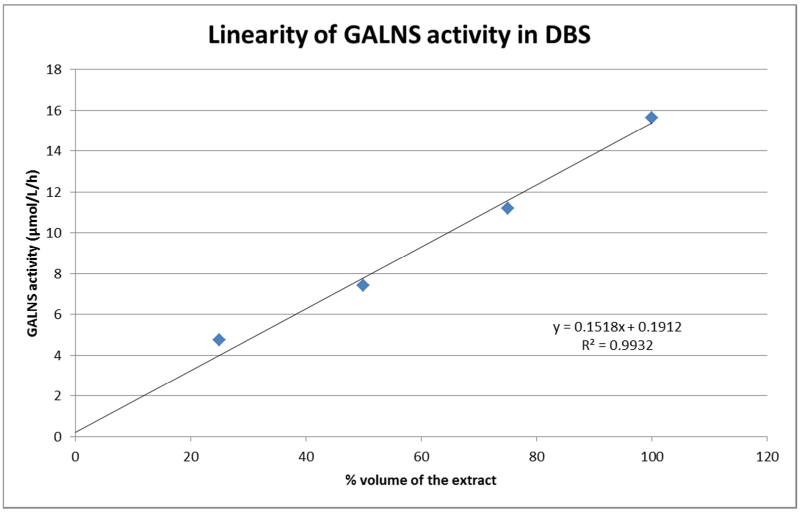

Supplement: S4 File — (PDF) [file pone.0131228.s004.pdf]
